# Supplementary material for: TDRD5 Is Required for Spermatogenesis and Oogenesis in Locusta migratoria
Source: Insects. 2022 Feb 24;13(3):227. doi: 10.3390/insects13030227 (PMC8953433; doi:10.3390/insects13030227)
Supplement: Supplementary file 1 [file insects-13-00227-s001.zip › insects-1597252-supplementary.pdf]

Supplementary Materials:

Table S1. Primers used in this study.

Gene name	Primer sequences (5'-3')	Purpose
LmTdrd5	F:CTCTGCAATTCAGTAATGGAA	RT-qPCR
	R:AAGCGTGGCAAAGGTTGTCTC	
LmVgA	F:CCCACAAGAAGCACAGAACG	
	R:TTGGTCGCCATCAACAGAAG	
LmVgB	F:AACGCCGACAGTGTTGGTATTC	
	R:ACCATCAGAAGTCGCTGGAAGT	
LmVgR	F:TTCAAGAGGCTGTCGGGTTCC	
	R:GCAGTCATGAGGTCGGTCTTCT	
LmACT	F:GCACCAAGAGCTTCATCCCT	
	R:CACGGCTCGTTCTTGTAGGT	
LmCREM	F:ATCCAGCCTAACCAGCAGTC	
	R:CCACAACCTGCAATGTCTGA	
LmQrich2	F:TCATCTCCCAGACCATCCGTAA	
	R:TGAATGCTGCTCCTCACCTTG	
β-actin	F:CGAAGCACAGTCAAAGAGAGGTA	
	R:GCTTCAGTCAAGAGAACAGGATG	
LmTdrd5	F:taatacgactcactatagggTCACAGAGGAAGAGGGGATG	dsRNA synthesis
	R:taatacgactcactatagggTTTCGTGGTGATGTTTCAGG	
GFP	F:taatacgactcactatagggGTGGAGAGGGTGAAGG	
	R:taatacgactcactatagggGGGCAGATTGTGTGGAC	

Abbreviations: dsRNA, double-stranded RNA; RT-qPCR, quantitative real-time polymerase chain reaction

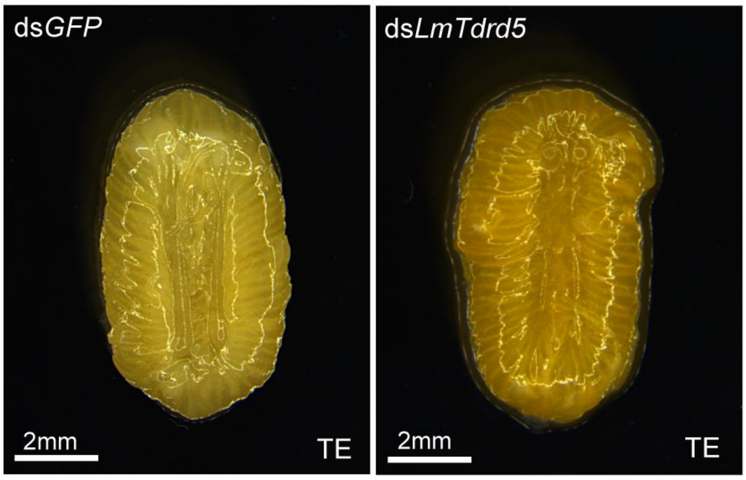

Figure S1. Phenotype of the testis from ds*GFP*- and ds*LmTdrd5*-treated males at ADD6.

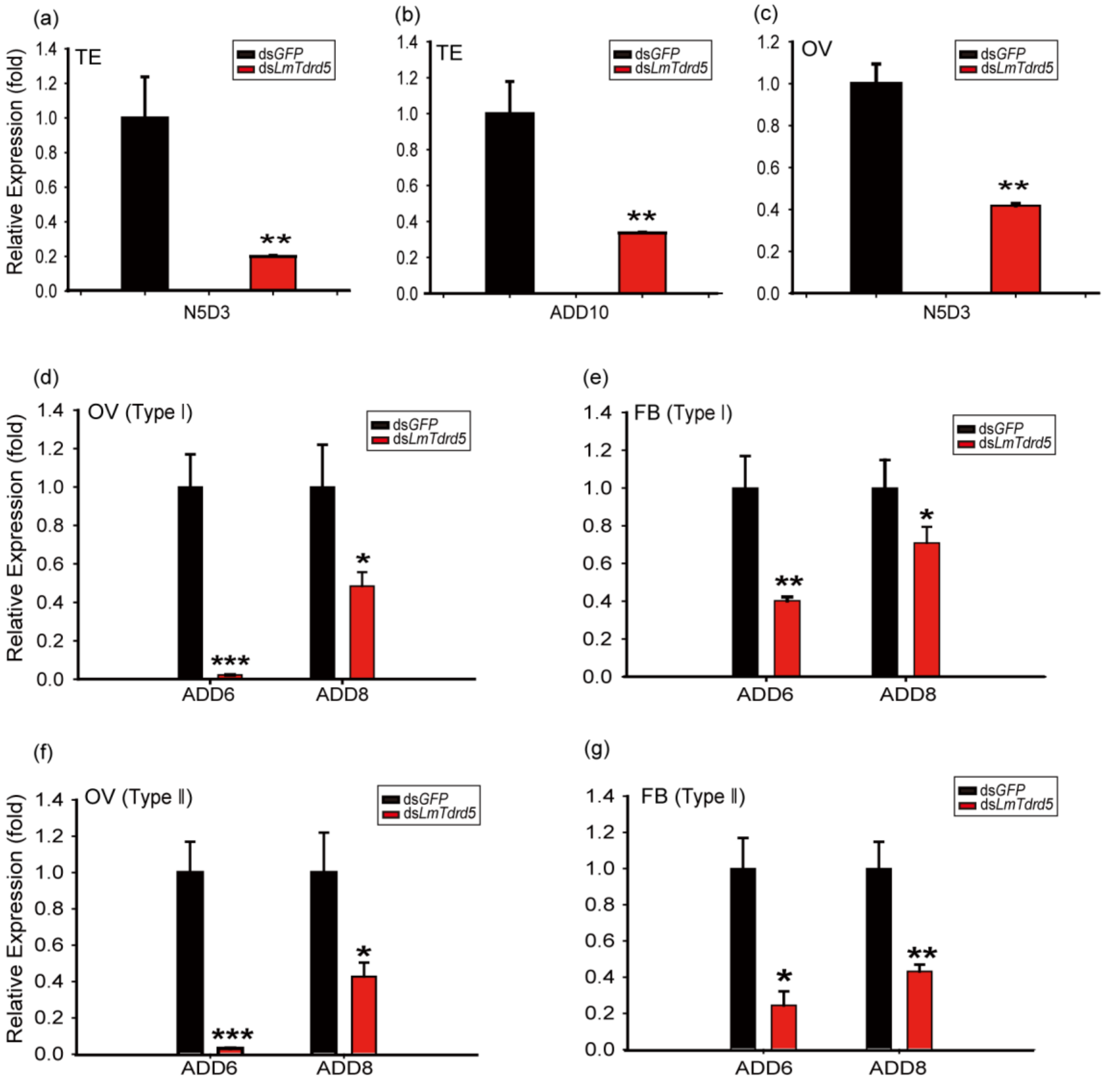

Figure S2. *LmTdrd5* RNA interference efficiency in the testes, ovaries, and fat bodies of ds*LmTdrd5*-treated males and females.

Experimental insects were injected with ds*LmTdrd5*, and the control group was injected with ds*GFP*. **(a, b)** *LmTdrd5* transcript levels in the testes determined by qRT-PCR at 48 h after dsRNA injection (N5D3, **a**) and ADD10 (**b**). (**c**) *LmTdrd5* transcript levels in the ovaries examined at 48 h after dsRNA injection (N5D3). (**d, e**) *LmTdrd5* transcript level in the ovaries (**d**) and fat bodies (**e**) of type I separately examined at ADD6 and ADD8. (**f, g**) *LmTdrd5* transcript levels in the ovaries (**f**) and fat bodies (**g**) of type II at ADD6 and ADD8. *LmTdrd5* expression was significantly reduced at all stages examined above. Statistically significant differences are indicated as follows: *$P < 0.05$; **$P < 0.01$; ***$P < 0.001$.

Supplemental File S1. The coding sequences of *LmACT, LmCREM and LmQrich2*.

>***LmACT***

ATGTCCAAGGACTGGCACTCGGGACACTTCAGCTGCTGGCAGTGCGACGAGTCGCTG
ACGGGCCAGCGCTACGTGCTGCGCGACGACCACCCCTACTGCATCAAGTGCTACGAGT
CCGTCTTCGCCAACGTCTGCGAGGAGTGCAACAAGATCATCGGAATCGACTCCAAGGA
CCTGTCGTACAAGGAGAAGCACTGGCACGAGGCGTGCTTCCTGTGCAACAAGTGCCG
CGTCTCCCTCGTGGACAAGCAGTTCGGCTCCAAGGGTGAGAAGATCTACTGCGGCAAC
TGCTACGACTCTCAGTTCGCCTCCAGGTGCGATGGCTGCGGCGAGATCTTCCGCGCAG
GCACTAAGAAGATGGAGTACAAGACACGCCAGTGGCACGAGAAGTGCTTCTGCTGCT
GCGTGTGCAAGACTCCAATAGGCACCAAGAGCTTCATCCCTCGTGAACAGGAGATCTA
CTGCGCAACTTGCTACGAGGAGAAGTTCGCTACAAGATGTGTCAAGTGCAATAAGATC
ATCACAAGCGGAGGTGTGACCTACAAGAACGAGCCGTGGCACCGCGAGTGCTTCACG
TGCACCAACTGCAACACGTCGCTGGCTGGCCAGCGCTTCACGTCCCGCGACGAGAAA
CCCTACTGCGCCGACTGCTTCGGCGAGCTGTTCGCCAAGCGCTGCACTGCCTGCTCCA
AGCCCATCACAGGCATCGGCGGCACGCGCTTCATCTCGTTCGAGGACCGCCACTGGCA
CAACGACTGCTTCATCTGCGCCATGTGCAAGACGTCGCTGGTGGGCCGGGGCTTCATC
ACCGACGCCGACGACATCATCTGCCCCGACTGCGCCAAGCAGAAGCTCATGTGA

>***LmCREM***

ATGGATGGAATGGTGGACGAAAACGGAACCGGTGCAACTGATCCCTTGGCTCATTCGC
CGGGGGACACGTCGTCACAAGTTGTTGTCACTTCGGTCCAATCAGTTATCCAGCCTAA
CCAGCAGTCTGTCATACAGACACCATCTAGTATACACCCTGTTCAACTTCAGAAAGGCA
ATGTAATTCTTGTGAGCAAGCCAAATTCTGTGATACAAACGACCCAAGGAAGTCTTCA
GACATTGCAGGTTGTGGAAACAGGCAGTGATGATAGTTTATCAAATGATGATGAATCCA
GCAAAAAAAGGAGAGACATATTGAGCAGGAGACCATCATACCGGAAAATACTAAATGA
TCTTGGTGGTGGTGAAATTGCAGTGATACCTGGCACAATTCAGATTGGCAGCCAAGGA
GAAACTGTGCAGGGTTTGCACACTCTCACCATGACTAATTCCTCAACAGGTGGTGCTAT
TGTACAATATGCCACACAGGGGCAAGATGGACAGTTCTTTGTTCCAGTTGTAACCCAG
GGAGGTGGACTTGGCAGTGGGCCAATAATTGCAGAGGATCAGGCCCGCAAAAGAGAG
ATGAGGTTATTGAAAAACAGGGAAGCTGCCAGAGAATGCAGGAGGAAAAAAAAGGA
GTACATCAAATGCCTGGAGAACAGAGTAGCTGTGCTAGAAAATCAAAACAAGGCCCTT
ATAGATGAACTGAAACAGTTAAAGGATCTTTATTGCCAACAAAAAACTGAGTGA

>*LmQrich2*

ATGCCATCAGAAACCCTTGTTCCATTACCAGCACTAATTGATTTTGCATTGGGGACTCCT
GAGATTGGGGCTGTTAATTTCAACATTTTACATTCTGTACTGCATGTTATAGTACAACAA
ACAAACTTGTATGAAACACAAGTTGAATTTAAAGGCCATGATGCTGAACGTCTTGAGA
CTTTAGTTAAACAGTCATCTCCCAGACCATCCGTAAGAATTCAGGAATACCAGTTACCT
GTTGTTACTGTAAAAGCATCAGTAGTAGAGCCTAGCAAGTCAGCAGAAGACACTACTG
AAGCAAAAGAAGAGCCACAAGGTGAGGAGCAGCATTCAGAAACTGAGCTTCAGACA
GTGGTATTAGTGGAATCAACTGGTGTAGAAGACTCTCCAGAAACAGATGAACATGATA
CAGCTAGACTCTCAAAACATGGATCACATCGGCAGACAAGACATTCAACAGGTGCAAA
AACGAGTGACCAAAGTGTCATTGTTAAAAAGGGGAGTTTTGAGAGACTTGAGAGAAA
GGTTGCCAAGATTCAAGCAGAATTGAAGAAGCTGGATGCACTTCCTACTAATGAAGAG
CTCATAAGACGTTCACGTGTATCAAGTGATTCTACACCGGTAAAGGATATGTGGCAGTA
CTTAAATCTCACAAAGAGATTGGATGCTGTTGAGGAGACCATTGAGAAGATAACGTCT

GTACTGCAACTTATAGCAAAGCAGATCGGTATGTCAACTATTTTAGGAGAGCCAGTGGA
CTCACTTGGGTCCCAGAGTGGTTCAAGCACTGATATGAATCCACTAGAGATCAAGGAT
GAAGCTGAACTTAAAGAGCGACTGATTAATGTTGAAAAGAGGTTATGGGATTTGGAAC
ATCGTGTACATTCCTCAGATTCATACATTCGTGGCTCTCGAGATGATGAGAGACATGGT
GAGGGATCTGGTGAGATACGTAGTGAGGGATCTGGTGAAATACCTGGCGGGAGCCCTA
GTCAGGGACATGGGAGCCCTGGTGAGAGACGTTTCCCTTTTGTTAACTATGCGGGACT
AACAACAGAACAGGCACTAGTTGCTCTGATAGGGGAGATGCGGCTTGTCCAGGATGA
AATCGAACTACTTTTCCAACAGCTTCATGATCATATAGCAGCCTCAGGAAACGAAACAA
TGGGTGGTGACTGTGCAAGGATCCTAGAGGAACTACAACAGAAGATGGAGGAACGTG
AGCAAGAAGCTTTCCAGCCATGTGCTAGAGTGAAAGATCTTGAAGATCGTATAACAAC
AAATACAACTCTGATACAAAACATGGAACTTAACTTTTCATCTCAAGTAGAAAATTTGC
AGCTCCAAATAGGGGATTTGGAGAAAGAACTGGGAGTGATAATGGAAAAATTTAATAC
TGATGAAGGAATGCAAGAGTCATCTTTACAGGATCTCTCAGGCCTCACTGAATTATACA
ACAAGATACAAACATTGCAAATTGAGATGGATAATGTGACCACTACAACAACACAGCT
TATGGATGACCGTGAAGAACGACAAATGCATATTAATGCACTGCTGGAGCAAATAGAG
CTCCTAAAGACAGTAAAAGCAGACAAAGAAGATCTGGAAGATGCACTTGCTGAGAAG
GCAGATCATGCTGCTGTGAATAGGAAGGTTTCACATGATCAATTTGAGGCTGCATGTAA
TGATCTTGCAAGTGGACTTGAAGATGCACTTTTGAAACTAAATGAGCAGAAAAAAATT
GTGGGAGGAAGCACTGAATGA

Supplemental File S2. Amino acid sequences of PIWI proteins from *L. migratoria*.
The RA/RG motif in red color
>LmPIWI1
MDGAGRRGRARGRARGQPPQQEGGVGRMHQARGPGEGPPHPAHPQPGIGRAARAAAT
MHARRPGEGPVQPPETFTTHETGGRALLRGATSRPGGAAGGDSERALLEKKMEELAIGG
DGNGAQLSVGRGVMRGRKVVSTEALKLRPEHLPTKKGTSGVRATVLTNYFRLETHTDW
RLFQYRVDFAPEEDNTFVRKALLRVHKKRLGGFIFDGTVMFTSSRLSPDPNEVLELTSERN
SDGAKIQILIKFVGDLALGDYHYLQFFNILMRKCLGSLKLQLVGRNYFDAAARISVKEYK
LELWPGYITSIRQHENAILMNSEINFKVMRQDTVLHIYTEICDKERDYQAAFKKVIVGVIV
MTPYNNRTYRIDDVDFTKTPSSKFPKKTGEEISYKDYFKQKYDLNIRGERQPLLVSRSKPR
ERRAGQAELVYLVPELCKMTGISDDMRANFHLMRALAEHTRVAPEARIHKLLQFNERLYR
EPEVVSSLTEWNMKLGRNLVDVEGRILPQEKIVQSANIKYDAGHDADWTRQLRSNPMFV
CCSLKNWAVISPTRCMRDAGSFVQTLQRAAGGMRINVPEPYYHEIPDDRQGTYVEALEY
VINSKNPQLIMCVVTNNRSDRYGAIKKKCCIDRAVPTQVMLAKNLASKGVMSIATKVAIQI
NCKLGGIPWTVEIPLSGMMTIGFDVCHDTTNKSRSYGALVASLDKQMSRYFSAVAPHASG
EELSNEMTANIIKALRKYQQVNQGSLPQRIFLYRDGVGEGQLHYVFEHEVALLKQRLQEV
YGSATFKLSVIIVTKRINTRLFLKQGNPPPGTVVDDVITNPSWYDFFIVSQSVRQGTVSPTA
YNVISDNSGLDADKMQRLTYKLTHLYYNWSGTVRVPAPCQYAHKLAYLVGQALHRPPQP
ALEDLLYFL

>LmAGO3
MEGIGRGGRGAKLLEALRAQQRRPGSEEPEGSQQSTEVKSGFSRGRGVLFAEQHAAPSQE
FTEAPSRDAPPVSGGRGHKLMALMAQQKSQTATPGPSLTAAGPAATGPSAAVAAAAAPPT
PAVRPVGRGRAALYEALRSMPGTSRGIPSPASAPTPAPTPKVSQTAVPVESLTRKMEQVEIE

RKPPVVFMGEQGERIPATANYVRLAVQPDKGVFEYEVRFTPTLDSKIIRSKILNSHSESIGRC
KTFDGVTLYLPMKLPEEVTTLTSNHPVDNSPVTTKIIFKRKKNLHECVHLYNVLFKRIMGI
LELARIGRDTFDPHAARVIPQHKLEVWPGYVTAVDEYEGGIQLCCDTSHRVLRTQTVLDVI
AEISSRNPAAFKDHIMKTVIGASVLTRYNNKIYRVDDIAWDRSPNDSFDTTSGDTITYCYY
YKKQYGLEIKDLRQPLLISKVKKKLQNQEKIEQLICLVPELCFMTGLTDEMRSDFKVMKDI
ATYTRVTPNQRQGSLKTFIERVMSNDRAKSLLEDWGLKLEPSTVELTARVVKNEDIIFGGN
RKVPGSQQAEWSGALSRNLVISAIDLMSWVLLRTARDLRYGNDFIDTMQKVGPQMGIQIA
QPSVVELRDDRTESYVRALRESIRPNVQIVVIICPTARDDRYSAIKRICCSEMPVASQVINSR
TLSKREKLRNIVQKIALQINCKLGGTLWAVQVPLDNLMVVGIDSYHDTLRKGSSVAAIVSS
LNKSVTRWYSQVSIQGPGQEWVDSLRACFISSLKKYHEVNHKFPDKIIVYRDGIGDGQLA
VCRDYEVPQFINCFPNINPSYSPKLTFIVCQKRINTRIFAQKNGSLDNPPPGTVMDNTITRRD
WVDFFLVSQNVRQGTVSPTHYVVIHDTSSMQADHIQRMTYKMCHLYYNWPGTVRVPAC
QYAHKLAHLIGQNVRKEHSDKLSDRLFFL

>LmPIWI2
MSSMMSGSIIGRTRARGRARGPATETTPSEPLTRTRTSGRGRTSAAMFDMTKLADTVPKVT
VPKPQAAPKRNVQRQTRVVEDSIITRPEGLSSKKGKSGTTVRMSTNYFKLTSITDFMLYQY
RVDFAPEEETTAAKIQLLKNHKEVLGGYIFDGSVMFTAMKLERDVMELYSTRETDGTKM
RITVRLVGVLSTGDPQYLQVFNILVRRCLQGLNLQLVGRHYFDAQSSVPIRNYSLELWPGY
ITSIRQHEDEILMCTEVTHKVMRMETVHDILRRVAQNNPHTFQEEFKKKVIGTIVLTDYSN
KTYRVDDVDFTVSPRNTFKRPSGETCYLEYYKNAYGIIIKDQQQPLLVSQPKQRDRRRGIN
NLIYLIPELCRTTGITDEMRSNFSLMRALAEHTRLGPATRQERLRKFQDRLRTTPQVQKDL
KQWNMSLSDELVTVPARVLDPEKIFLQKAQCTVGQKNDWTQEVKSRGLLSAPVLKKWGI
LCPNSQLRACKEFELTLQKAAAAIDFKVEDGVVYSVQEEQGIAGYIQALDYLLSSFTPQIIL
IILQNKRADRYNALKRRCCVDRAVATQVVLLKNTEGKNIFSVASKIVIQMCCKIGGAPWSI
EIPLSGLMVVGFDVYHDTASRGVSIGALVASMNKALSRYFSAVSYQHSGEELSNELSINMC
KALQKFQSMNGSLPERIIIYRDGVGEGQIPFVMEHEVELLKGRLKEIYGGRHAKMTFIIVT
KRINSRVFYNKQNPPPGTIVDDVITLAQRYDFFLVSQSVRQGTVSPTSYNIIYDNVGLEPDK
IQRLTYKLTHMYFNWSGTVRVPAPVQYAHKLAFLVGQSLHTRPSNALEDFLYFL
